# Metabolic Pathways as a Novel Landscape in Pancreatic Ductal Adenocarcinoma

**DOI:** 10.3390/cancers14153799

**Published:** 2022-08-04

**Authors:** Ahmad Ali, Ugo Chianese, Chiara Papulino, Antonella Toraldo, Mawada Elmagboul Abdalla Abakar, Eugenia Passaro, Rosario Cennamo, Nunzio Del Gaudio, Lucia Altucci, Rosaria Benedetti

**Affiliations:** 1 Department of Precision Medicine, University of Campania “Luigi Vanvitelli”, 80138 Naples, Italy; 2 AORN Chirurgia d’Urgenza, Ospedale Cardarelli, 80131 Naples, Italy; 3 Biogem Institute of Molecular and Genetic Biology, 83031 Ariano Irpino, Italy

**Keywords:** PDAC, metabolism, glucose, amino acids, lipids, immune response

## Abstract

**Simple Summary:**

The survival of organic systems is dependent on metabolic conditions, necessary for the proper functioning of all biological processes. Cancer takes advantage of altered bioenergetic circuits to improve its chances of growth, and pancreatic ductal adenocarcinoma is no exception. In this review, we describe the metabolic features of pancreatic ductal adenocarcinoma and discuss how this dependency could be exploited as a weakness for clinical interventions.

**Abstract:**

Metabolism plays a fundamental role in both human physiology and pathology, including pancreatic ductal adenocarcinoma (PDAC) and other tumors. Anabolic and catabolic processes do not only have energetic implications but are tightly associated with other cellular activities, such as DNA duplication, redox reactions, and cell homeostasis. PDAC displays a marked metabolic phenotype and the observed reduction in tumor growth induced by calorie restriction with in vivo models supports the crucial role of metabolism in this cancer type. The aggressiveness of PDAC might, therefore, be reduced by interventions on bioenergetic circuits. In this review, we describe the main metabolic mechanisms involved in PDAC growth and the biological features that may favor its onset and progression within an immunometabolic context. We also discuss the need to bridge the gap between basic research and clinical practice in order to offer alternative therapeutic approaches for PDAC patients in the more immediate future.

## 1. Introduction

**Figure 1:**
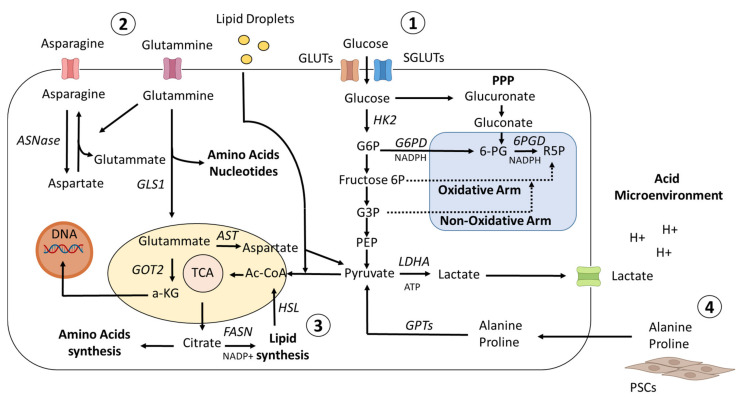
Metabolic landscape in PDAC. (**1**) To promote glucose uptake in PDAC cells, KRAS and HIF1 upregulate the GLUT family of genes and other genes associated with glycolysis. While a portion of the glycolytic cascade is used to fuel oxidative phosphorylation and the production of ATP, or alternatively to promote lactate, which helps to create an acidic microenvironment, another branch of the process is directed toward the PPP pathway to provide precursors for nucleotide and amino acid biosynthesis. (**2**) Cellular redox homeostasis and energy generation are both regulated by amino acid metabolism. Glutamine is transformed into glutamate and aspartate, which are then transported to the mitochondria to maintain redox balance. (**3**) Citrate is shuttled from the mitochondria into the cytoplasm to stimulate the de novo lipid synthesis pathway, and at the same time, redox processes are balanced by NADPH–NADP^+^ conversion. This process activates the lipid synthesis pathway. In addition, exogenous lipid intake is boosted to meet the need for nutrients for rapid proliferation. (**4**) Different metabolites/nutrients, such as Ala and Pro, generated from collagen degradation or PSC secretion and transformed into pyruvate, are supplied to PDAC cells by the tumor microenvironment.

The incidence of pancreatic ductal adenocarcinoma (PDAC) is increasing in recent years and is expected to become the second-leading cause of cancer death by 2030 [1]. PDAC is an aggressive tumor, often diagnosed at an advanced stage due to the lack of symptoms [2,3]. PDAC is not always treatable with surgery and, in fact, only 20% of patients have resectable disease [4,5]; similarly, chemotherapy treatments are poorly effective. In a cohort of 136 patients, 74.3% experience relapse within one year, while 25.7% was found to recur within the first six months of surgery [6]. Next-generation sequencing (NGS) technologies analyzing the mutational profile of PDAC patients recently identified mutations occurring at early or late stages of the disease [7]. *KRAS*, detected in 90% of cases as mutated and/or amplified [8], is chronologically the first mutated gene in pancreatic intraepithelial neoplasia (PanIN) lesions [9], precancerous stage. The second-most-commonly mutated gene in PDAC is *TP53*, predominantly presenting missense mutations [10] associated with a very poor outcome [11]. Another common mutant gene in PDAC is *CDKN2A*, encoding a cyclin-dependent kinase inhibitor, resulting in loss-of-function alterations; a lower degree of differentiation of PDAC cells was related to a more rapid *CDKN2A* degradation [12]. *SMAD4* mutations occur in late stages of PDAC and seem to be linked to its inactivation in approximately 50% of pancreatic cancer cases, promoting tumor growth and metastasis [13] and representing an increased risk factor for overall survival [14]. The main classification of pancreatic cancer distinguishes between exocrine and neuroendocrine tumors [15,16]. The vast majority of cases (95%) are exocrine pancreatic cancers, including PDAC, which accounts for more than 90%, and acinar cell carcinoma, which makes up 1–2%. Neuroendocrine cancers are rare, comprising less than 5% of all pancreatic cancers [17]. PDAC is often associated with other metabolic comorbidities, such as obesity and diabetes, which occur in 15–35% of PDAC patients and are considered risk factors [18,19,20]. Algorithms and prediction models were developed to identify high-risk patients among a large number of obese and diabetic patients [21]. Some antidiabetic medications, such as metformin, may decrease the risk of PDAC [22], while others, including insulin, are associated with an increased risk [23]. The fact that PDAC exhibits a marked metabolic phenotype (Figure 1) suggests that the metabolic environment may play a key role [24]. PDAC has high energy requirements, which are met through the rewiring of cell metabolism. Nutrients are, therefore, consumed to provide energy, ensure biosynthesis, and minimize oxidative stress. PDAC exploits metabolic pathways to sustain rapid cell proliferation [25], thereby depleting major nutrients in the tumor microenvironment and adversely affecting other cell types, in particular, immune, acinar, and ductal cells [26]. PDAC cells are surrounded by immune cells, stellate cells, cancer-associated fibroblasts, and extracellular matrix (ECM) [27,28]. According to their metabolic profile, PDAC cells are divided into three metabolic subtypes: slow proliferating, glycolytic, and lipogenic [29]. Metabolic plasticity also makes a major contribution to cancer heterogeneity [30]. Single-cell RNA sequencing analysis identified distinct types of ductal cells according to their gene expression profiles based on PDAC heterogeneity [31]. A common subtype of ductal cells was found in both healthy and cancerous tissues, while a second subtype, showing altered energy distribution, was found to reside in PDAC tumors [32]. PDAC is also associated with inflammatory states, which contribute to its progression [33]. Furthermore, inflammation has been linked to the immunometabolic context, since pro-inflammatory stimuli may induce a metabolic switch in hematopoietic cells, increasing aerobic glycolysis similarly to the Warburg effect [34], the well-known shift to aerobic glycolysis (lactate production) in the presence of oxygen [35]. Single-cell sequencing suggests that macrophages, T cells, and fibroblasts are highly heterogeneous within the tumor microenvironment [36] and are strongly affected by its metabolic context [37]. In vivo mouse experiments revealed that macrophages exhibited elevated glycolysis and that macrophage-specific deletion of GLUT-1 reduced tumor burden by increasing natural killer and CD8^+^ T cell activity and suppressing the inflammatory state [38]. Glutamine antagonists are also reported to induce a change in the antitumor immune response by converting a “cold” tumor microenvironment into a “hot” one, eliciting significant responses to anti-PD1 therapy, a cell surface protein involved in the suppression of the immune system [39]. Taking all these findings together, it seems clear that metabolites play a crucial role in homeostasis and tumor progression. The analysis of metabolic turnover in the tumor microenvironment is, therefore, key to defining the energy phenotype and metabolic landscape of PDAC.

## 2. Metabolic Reprogramming of the Main Energy Pathways in PDAC

### 2.1. Activation and Maintenance of Glycoltyc Flux

In PDAC, the expression of glycolytic genes is regulated at both the transcriptional and post-transcriptional level via oncogenic KRAS [40]. KRAS signaling plays a crucial role in regulating transcription of both glucose transporters (GLUTs) and key glycolysis genes [41]. Glucose’s need of the PDAC system seems to be due to the expression of facilitated GLUTS and sodium–glucose transporter (SGLTs); PDAC tumors showed both increased pyruvate carboxylation and glucose oxidation via pyruvate dehydrogenase in vivo [42]. PDAC progression is induced by the activation of mutant KRAS, resulting in an increase in GLUTs, such as GLUT-1, from low- to high-grade dysplasia. Oxygen is related to GLUT-1 expression through hypoxia-inducible factor 1 alpha (HIF-1α). In patients with low-expression levels of GLUT-1, neoadjuvant chemotherapy, such as TS-1, showed better therapeutic response and better prognosis than in those with higher GLUT-1 expression levels [43]. PDAC tumor biology relies on hypoxia and HIF1α signaling to control tumor-promoting stromal programs, which facilitate progression and tumor cell invasiveness [44]. Hypoxia-activated stromal cells contribute to the invasive growth of PDAC cells by releasing soluble proteins, such as MMP10, and enhance the levels of inflammatory and angiogenetic factors, including IL1α, TIE family members, and VEGF-A. MMP10, the main stromal protein driving EMT in tumor cells [45], is reported as a stellate cell product [46], [47]. IL1α was shown to be released by both stromal cells and PDAC cells, thus, promoting tumor growth [48] in an autocrine manner [49] and stimulated the fibrotic component [50]. TIE1 upregulation and increased TIE2 transcription in hypoxic stellate cells are crucial for the remodeling and maturation of tumor vasculature [51], forming a complex with angiopoietins and sustaining TIE2 signaling in contacting cells [52]. Altered levels of VEGF-A found in PDAC indicate an imbalance in normal angiogenetic processes [53]. In particular, the high extracellular matrix component associated with vasculature collapse resulted in an increased hypoxic environment, partly explaining the low efficacy of antiangiogenic drugs in this cancer [54] and the inefficient delivery of chemotherapeutic agents [55], thus, emphasizing a recently described stroma-targeting therapy that aims to reduce the stromal component to improve target achievement [56]. According to the transcriptomic profiles of PDAC patients, ubiquitin specific peptidase 25 (USP25) depletion was linked to decreased levels of HIF-1α, GLUT-1, and glycolysis signaling. This suggests that the USP25 complex deubiquitinates and stabilizes the HIF-1 α transcription factor from a mechanistic point of view [57]. SGLTs also play a functional role in glucose uptake, since the selective inhibition of SGLT2 in mouse models of pancreatic cancers led to a decrease in glucose uptake [58]. Furthermore, the hypoxic environment is essential for maximizing energy yield and biomass production, which are ensured by the lack of oxygen, which promotes conversion of pyruvate into lactate, by Lactate Dehydrogenase A [59]. In this way, ATP is generated and an increased amount of lactic acid is released outside of the cell, acidifying the microenvironment and, in turn, facilitating PDAC progression [60]. Monocarboxylate transporters (MCTs), which transport lactate, are abundantly expressed in PDAC [61]. MCT1 and MCT4 regulate lactate efflux through KRAS-dependent signaling, releasing intracellular accumulated lactate and maintaining intracellular pH [62]. This process facilitates the oxidation of nicotinamide adenine dinucleotide (NADH) to NAD^+^, a cofactor for oxidizing glyceraldehyde 3-phosphate and driving glycolysis [63]. The glycolytic shift meets the energy demands required for tumor growth, as well as supplying the building blocks for biochemical reactions and intermediates [64]. MUC1 and MUC13 transporters also stabilize transcription of HIF-1α during hypoxia conditions and induce the expression of glycolytic genes [65] associated with poor survival rates in PDAC patients [57]. In contrast, CD147 works as a chaperone for the membrane localization of MCT1 and MCT4, both expressed in PDAC cells [66]. Whether the interaction between CD147 and MCT is related to PDAC progression has not yet been determined [67]; however, depletion of MCT4 reduces cell viability, whereas depletion of CD147 affects tumor growth in xenograft models [62,68]. As regards the glycolytic flux of anabolic pathways in PDAC, the pentose phosphate pathway (PPP) is a branch of glycolysis that directs glucose flux to oxidation and regulates NADP and nucleic acid synthesis, which ensure fatty acid (FA) production and cell survival under stress conditions [69]. According to a metabolomic analysis of PDAC, the adaptation to acidosis status increases glucose and decreases glycolysis, driving a shift to PPP [60,70]. PPP occurs in two different ways: oxidatively and non-oxidatively. The oxidative arm transforms glucose 6-phosphate into ribulose-5-phosphate and CO_2_, which are essential for maintaining redox equilibrium under stress conditions [71]. The non-oxidative branch produces glycolytic intermediates, resulting in the production of sugar phosphate, an important precursor for amino acid synthesis, ribose-5-phosphate, which is needed for nucleic acid synthesis [72]. Furthermore, oncogenic KRAS selectively activates non-oxidative PPP, possibly via the induction of genes involved in the non-oxidative arm, such as ribulose-5-phosphate isomerase (RPIA) [73]. Low expression levels of RPIA deficits result in reduced KRAS-driven signaling in PDAC cells, indicating the importance of non-oxidative PPP in metabolic function [40]. Growing evidence suggests that non-oxidative PPP contributes to gemcitabine resistance in PDAC and that reduced expression of transketolase is associated with higher gemcitabine sensitivity in PDAC patients, strengthening the therapeutic potential of targeting non-oxidative PPP [65]. Post-transcriptional processes, such as those modulated by microRNAs, are also thought to play an important role in PDAC progression [74]. microRNAs are linked to the regulation of glycolysis in PDAC; the tumor suppressor miR-124 regulates MCT1 [75], resulting in increased intracellular pH that reduces the acidic environment and decreases PANC-1 cell proliferation. miR-135 was found significantly overexpressed in PDAC patient samples compared to normal tissue and, notably, was associated to a metabolic alteration. miR-135 accumulation during glutamine deprivation has been observed, promoted by mutant TP53. Specifically, miR-135 targets phosphofructokinase-1, inhibiting aerobic glycolysis and promoting TCA cycle [74]. Some studies have already been conducted on the potential role of miRNA as biomarkers of PDAC. miRNA-483-3p and miRNA-21 were found to be significantly higher from blood plasma in PDAC compared to healthy controls and related to advanced-stage disease [76,77]. Further functional studies on miR-124 may lead to new therapeutic strategies for PDAC [75].

### 2.2. Amino Acids as an External Energy Resource

The PDAC phenotype is also triggered by the rewiring of amino acids, contributing to the metabolic profile of PDAC by regulating cell proliferation, invasion, and redox homeostasis [78]. In cellular hemostasis, glutamine is a multifunctional amino acid that serves as a key energy source [79]. The biological activities of glutamine range from providing energy to stabilizing reducing agents, contributing to the biosynthesis of purines and pyrimidines, and its involvement in PDAC has been recognized [80,81]. PDAC cells can compensate for the increased metabolic demand either by increasing glutamine production or by increasing glutamine uptake from the environment, thus, reducing glutamine levels in blood serum, despite the abundance of fibrotic cells in the pancreas [82]. Glutamate–Ammonia Ligase (GLUL), the enzyme responsible for de novo production of glutamine, was found elevated in PDAC [83]. Although the cause of this increase is not completely clear, CRISPR/Cas9 ablation of GLUL in PDAC mouse models reduced tumor growth [83]. Metabolic niches also contribute significantly to cancer development and progression. Autophagy plays a pivotal role in supporting the growth of PDAC through fibroblasts [84]. Autophagy allows fibroblasts to break down misfolded proteins and ECM, releasing large quantities of amino acids into the microenvironment [85]. In addition, circulating macromolecules enter PDAC cells using the Na+-dependent glutamine transporter SLC1A5, in the case of glutamine, or via macropinocytosis/micropinocytosis, for proteins, a mechanism linked to the growth of cancer cells expressing oncogenic KRAS [86,87,88,89]. Micropinocytosis inhibitors were found to interfere with this ability in MIAPaCa2 cells, a PDAC model [90]. Glutamine intake is converted into glutamate to feed a complex network of enzymes and intermediates. PDAC utilizes glutamate to activate the tricarboxylic acid (TCA) cycle and electron transport chain after its conversion into alpha-ketoglutarate (αKG) in mitochondria; notably, αKG acts as an epigenetic factor [91]. αKG may also function in a TCA-independent manner by acting as a cofactor for dioxygenases [91], controlling gene expression, DNA methylation, and DNA damage reactivity [92]. Similar to glutamine, alanine is also required for metabolic homeostasis in PDAC and is derived from pancreatic stellate cells (PSCs) [93]. Several studies have investigated the unidirectional channeling of alanine between PSCs and PDAC [93,94]. SLC38A2 activity facilitates alanine uptake, although other transporters have been identified, including SLC1A4 [93]. PDAC cells also express the mitochondrial isoform of glutamic-pyruvic transaminase ALT2 for de novo synthesis and alanine utilization. The ratio between aspartate transaminase AST and alanine aminotransferase ALT was used to predict poor prognosis and response to gemcitabine/nab-paclitaxel treatment in PDAC patients [95]. In co-injection xenograft models, the beneficial support provided by stellate cells was disrupted by targeting SLC38A2, causing significant tumor regression in PDAC and affecting cytosolic alanine internalization and concentration [93]. PDAC can also use proline as a fuel source and this energy comes from collagen that is largely found in the ECM [96]. Proline degradation by the mitochondrial enzyme PRODH1 is an active factor in PDAC cell proliferation, both in vitro and in vivo [96], indicating that ECM is an important nutrient reservoir for cancer cell metabolic flexibility. Some context-specific metabolic mechanisms have also been described for PDAC, such as the TP53-mediated overexpression of SLC1A3, an Na^+^/K^+^/H^+^-dependent aspartate/glutamate transporter, which enables the aspartate metabolism to maintain cancer cell survival and tumor growth under conditions of glutamine starvation [97]. By perturbing glutamine metabolism, redox homeostasis proteins are deregulated, leading to reactive oxygen species ROS accumulation, which then leads to a cellular redox imbalance facilitating PDAC cell apoptosis [98]. Pharmacological and genetic targeting of nicotinamide phosphoribosyltransferase (Nampt), a key redox enzyme, inhibited cell growth and survival of PDAC cells in vitro and in vivo [99]. Other findings link amino acids with cell fate. KRAS-driven PDAC mouse models were less responsive to a depletion of serine and glycine [100]. Cysteine depletion induced ferroptosis in *KRAS/TP53* mutant pancreatic tumors in mice, and the disruption of amino acid pathways was able to enhance gemcitabine chemosensitivity in drug-resistant PDAC [98,101]. Ferroptotic damage can result in the release of damage-associated molecular pattern molecules, which can lead to inflammation [102].

### 2.3. Fatty Acids Contribute to PDAC Progression

Epidemiological studies correlated PDAC with dyslipidemia [103], showing an altered biosynthesis of cholesterol and other lipids in murine PDAC cells [104,105,106,107]. Lipogenic enzymes are frequently overexpressed in PDAC, supporting their potential contribution to tumor growth [108]. Alanine from PSCs can be taken up by PDAC cells and used for FA biosynthesis. Serum FA synthase (FASN) levels are, in fact, generally higher in PDAC patients [109] as a result of SREBP1 activity [110] and are associated with lower survival than in patients with low FASN expression and with poor response to gemcitabine [111,112]. Once again, driver mutations in KRAS and loss of function in TP53 reprogram metabolism accelerate cholesterol biosynthesis and uptake [40], mediating metabolic plasticity via SREBP1-dependent regulation of transforming growth factor-β expression involved in PDAC differentiation [105]. Oncogenic KRAS regulates hormone-sensitive lipase (HSL) to control metabolism by regulating lipid storage and utilization (specifically through suppression of HSL expression), leading to lipid droplet (LD) accumulation and priming tumor cells for invasion [113]. Perilipins constitute the major proteins resident on LD surface controlling intracellular lipid homeostasis [114,115]. Perilipin 2 (PLIN2) was found overexpressed in a cohort of 181 PDAC patients [116] and was associated with poor MFS, DFS, and OS rates, as well as with poor prognosis. Further investigations using an in vivo mouse model showed that exposure of pancreatic β cells to fatty acids stimulated PLIN2 expression, impacting on cellular stress, whereas its ablation prevented fatty-acid-induced TG accumulation [115], mitigating stress and leading to a significant improvement in hyperglycemia [117]. Notably, PLIN2 is expressed in other cell types, such as monocytes and macrophages [118], where its expression was positively correlated with LGALS9 in PDAC; this protein converts polarized macrophages into an M2 phenotype, leading to the inhibited secretion of T-cell cytokines [119]. These findings suggest that PLIN2 might participate in immunomodulatory effects by regulating tumor-associated macrophages in the tumor microenvironment [120]. A high-fat diet was able to ameliorate mutated KRAS activity, increasing fibrosis and enhancing PDAC progression in a mouse model [121], and a recent study with an in vivo mouse model showed a causal and positive correlation between obesity and early PDAC progression, identifying altered beta cell expression of cholecystokinin (Cck) in response to obesity and defining islet Cck as a promoter in oncogenic KRAS-driven PDAC [122]. LDs are recognized as important regulators in cancer; these dynamic intracellular organelles are used for cellular storage of lipids, such as triacylglycerol and cholesterol ester [123]. Lipids can, thus, be catabolized by lipolysis via lipases to liberate free FAs [124], causing increased FA oxidation and oxidative metabolism, which drives tumor cell invasion. Low-density lipoprotein receptor (LDL-R) is highly expressed in PDAC and is associated with increased PDAC recurrence [125]. LDL-R increases cholesterol uptake, while its inhibition reduces proliferation, affecting ERK1/2 survival pathway, and sensitizes PDAC cells to chemotherapeutic drugs, favoring tumor regression [125]. Interestingly, mutated KRAS is able to control the sequestration of extracellular unsaturated FAs [126]. ACSL3 activity, a protein-coding gene for a member of Acyl-CoA synthetase long-chain family, has been linked to KRAS-mutated tumors [127] and associated with the retention of extracellular unsaturated FAs by converting them into esters that remain confined in PDAC cells [128,129]. Serum lipid depletion or ACSL3 inhibition decreased tumor cell proliferation, provoking a rebound effect due to lipid restriction that was balanced by increased autophagic flux, in both in vitro and in vivo models [130]. Notably, combining lipid depletion with autophagy inhibitors induced the most potent effect, with arrest of PDAC proliferation and increased apoptosis [130]. Recently, metabolomic profiles clarified key aspects of the metabolic signature of pancreatic cancer stem cells (PCSCs) originating from PDAC cells, revealing a fundamental role for the pyruvate–malate cycle and lipid metabolism in their survival [131]. While lipidomic analysis suggested a strong induction of long-chain FAs and accumulation of LDs mediated by ELOVL5, a fatty acid elongase, other data highlighted cardiolipin acyl-chain composition as pivotal in PCSCs [132]. Changes in cardiolipin composition have an impact on enzymes involved in the respiratory process and integrity of the inner membrane [133,134], indicating that cardiolipin plays a critical role in oxidative phosphorylation. A comprehensive investigation on serum lipids of 830 PDAC samples by mass spectrometric determination revealed statistically significant differences between PDAC patients and healthy controls [135]. While a lysophosphatidylcholine LPC 18:2 was positively correlated with survival, Cer 36:1, Cer 38:1, Cer 42:2, PC 32:0, PC O-38:5, and SM 42:2 were inversely correlated, suggesting their potential role as prognostic biomarkers. Other data in PDAC tissues by MALDI-MSI analyses indicated that LPC (16:0, 18:1), as reported for other LPCs [136,137], and DAG 36:2 were decreased, while PC 32:0, SM d36:1, and SM d42:3 were increased [138]. Glycerophospholipid and sphingolipid metabolism pathways were also found dysregulated in PDAC [138]. Regarding lipid saturation degree, polyunsaturated phosphatidylcholines were reduced in serum of PDAC [139]. It is tempting to speculate that this altered profile might reflect apoptotic resistance in PDAC, given that polyunsaturated FAs, via peroxidation, act as substrates for ferroptosis in cell membranes [140,141].

## 3. Immune Cells and Metabolic Response in PDAC Microenvironment

**Figure 2:**
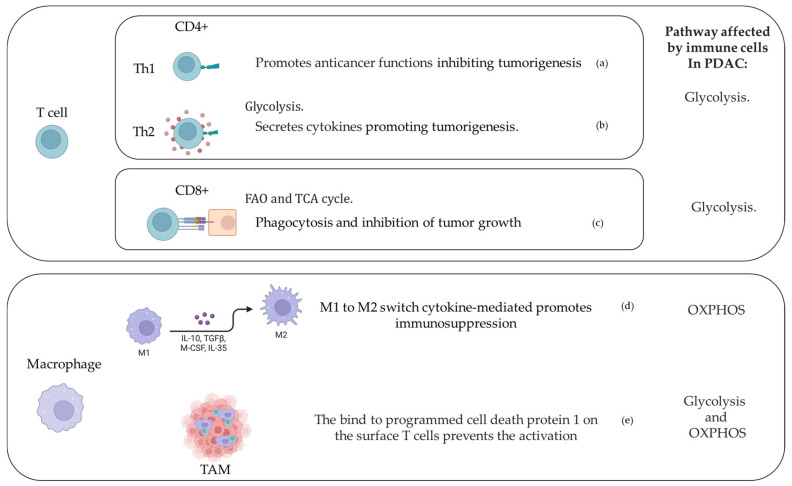
T-cell and macrophage activity in the PDAC-conditioned environment. (**a**) [154,169]; (**b**) [171]; (**c**) [148,172]; (**d**) [164]; and (**e**) [173].

Immune cell functionality and metabolism are closely linked and are able to influence each other [142]. In recent years, several studies provided compelling evidence that changes in cellular metabolism affect immune cell function (Figure 2), which, in turn, impacts cell metabolism [142,143] due to competition for nutrients. The high energy demands of tumor cells cause nutrient depletion, resulting in decreased rates of glycolysis in tumor-infiltrating lymphocytes [144,145]. The imbalance in metabolic profile and chronic inflammation can trigger autoreactivity and ultimately disrupt protective immunity. Several immunosuppressive cytokines were found to cause tumor development by impairing cytotoxic and helper T cells [146]. The acid environment created by lactic acid levels inhibited cytotoxic T cell function, promoting tumor growth [147]. In addition, immunohistochemistry analysis detected an increase in CD8^+^ and CD4^+^ T cells, leading to a better outcome in PDAC patients [148,149]. In PDAC, CD8^+^ T-cell activity is altered due to degradation via MHC-1, member of the histocompatibility complex, [150], while inhibition of autophagy restores surface levels of MHC-1, enhancing anti-tumor T-cell response, improving [151] clinical outcomes, increasing the survival rate for PDAC patients [152]; the effect observed in CD4^+^ T cells depends on their subtype differentiation [153]. T-helper 1 cells contributed to a positive clinical outcome via IFN-γ and TNF-α production, promoting anticancer activities through cytotoxic T-cell response [154,155]. Interestingly, insulin receptors are expressed on activated CD4^+^ T cells and can contribute to reshaping the adaptive immune system by regulating T-cell metabolism [156]. Induced knockdown of insulin receptors led to a reduced glucose metabolism and cytokine production in T cells [157]. Glutamine may also act as a regulator of effector and regulatory T-cell (Treg) balance in the PDAC microenvironment, reducing Th17 and Th1 cells and promoting the development of Tregs [158]. The production of αKG by glutamate dehydrogenase promotes cancer growth and interferes with immune response, acting as an anaplerotic intermediate in the TCA cycle and providing nitrogen for non-essential amino acid synthesis [81]. T cells need arginine and tryptophan for activation to generate memory T cells by switching from glycolysis to oxidative phosphorylation, thus, promoting tumor arrest [159]. PDAC is thought to be fueled by a feed-forward mechanism, in which amino acid intermediates support cancer growth by depleting arginine and tryptophan, inhibiting T-cell proliferation and promoting Treg differentiation [160]. Indeed, Tregs can inhibit immune responses mediated by T cells, as described in a study involving a total of 100 patients with PDAC [161]. The authors evaluated the prevalence of Tregs in peripheral blood mononuclear cells from patients in relation to their clinical outcomes and showed that the percentage of Tregs in the patients with PDAC was significantly lower than in healthy volunteers. Additionally, numbers of mast cells from 103 patients with PDAC and 10 patients with a normal pancreas were investigated about their distribution PMID: 21167541 [162]. Results showed a zone-specific distribution of mast cells in PDAC, highlighting the importance of invasive front in the prognosis of patients with PDAC after curative resection. Dendritic cells are an integral part of the PDAC tumor microenvironment, characterized by a reduced number compared to the healthy condition, which impacts antigen presentation and contributes to immune tolerance [163]. Macrophages have also been linked to the immunometabolic context in PDAC. In response to the environment conditioned by PDAC, macrophages switch from the pro-inflammatory M1 to the anti-inflammatory M2 phenotype [164,165]. M1 macrophages showed an enhanced glycolytic and lipolytic activity [166], promoted by fructose-2,6-bisphosphatase enzyme [167]. Furthermore, when PPP is inhibited, macrophages switch toward an anti-inflammatory state, increasing TCA cycle and FA oxidation [168,169,170].

## 4. Clinical Perspectives

**Figure 3:**
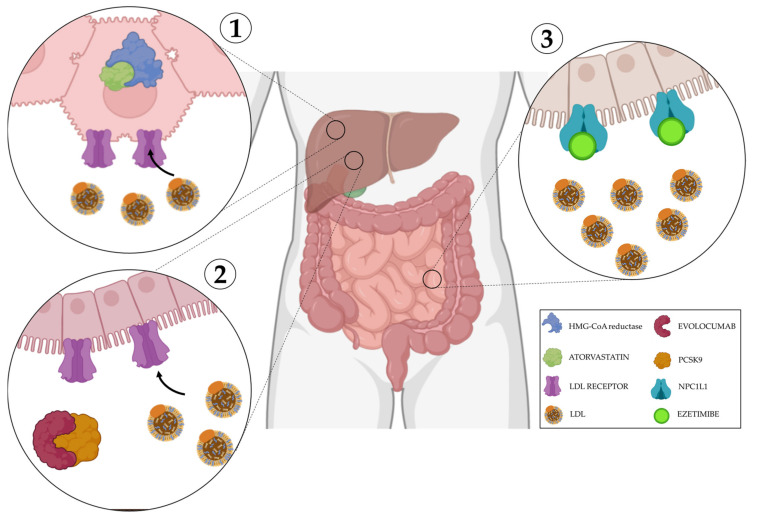
Mechanisms of action in cholesterol disruption. (**1**) Atorvastatin is a competitive inhibitor of HMG-CoA reductase, its inhibition decreases de novo cholesterol synthesis and increases expression of LDL receptors, removing LDL from the blood. (**2**) Evolocumab blocks PCSK9, a protein responsible for the breakdown of LDL receptors; this allows their overexpression facilitating LDL uptake from the blood. (**3**) Ezetimibe selectively inhibits the intestinal absorption of LDL.

Over the last decade, it has become clear that rapidly proliferating systems, such as cancer, use metabolism to facilitate cell survival and maintain growth [174]. Metabolic plasticity contributes to PDAC heterogeneity and although not all metabolic dependencies of pancreatic tumors have been revealed, some distinct phenotypes, such as the glycolytic and lipogenic subtypes, have been identified. As a consequence, potentially innovative strategies to treat patients with PDAC are based on glycolytic and glutamine inhibitors, which have shown efficacy for the glycolytic subtype, while the lipogenic phenotype is more sensitive to inhibitors of lipid biosynthesis. Currently, PDAC trials are mainly focused on immunotherapy and chemotherapy [174] and there are still few trials investigating cell metabolism. Here, we reported the clinical trials focused on metabolic evaluation in PDAC (Table 1), such as NCT05132244, that will investigate the feasibility to monitor and manage glucose levels in people with PDAC and the related impact. NCT04245644 aims to understand if regular use of statins and metformin can increase the rate of disease-free survival and overall survival in PDAC participants, before diagnosis, after surgery, and in a neoadjuvant treatment setting, and possible use as chemoprevention, while a large randomized study of 528 participants (NCT03504423) is evaluating the effect of CPI-613 (devimistat), a pyruvate dehydrogenase inhibitor, to determine its efficacy and safety in patients with metastatic PDAC. The aim of another study (NCT02201381) is to assess the effectiveness of a regimen of metabolic treatments for patients in order to determine the relationship between the degree of response and changes in biochemical markers. In a cohort of 207 participants, metformin, atorvastatin, doxycycline, and mebendazole will be administered to evaluate the effectiveness of heterogeneous classes of drugs. Pharmacologically, metformin improves insulin sensitivity and the oxidative disposal of glucose and lactate. NCT04862260 will investigate the effect on cholesterol disruption also in metastatic PDAC patients (Figure 3). Statins lower cholesterol by inhibiting HMG-CoA reductase, the rate-limiting enzyme of the metabolic pathway, producing cholesterol and other isoprenoids, thereby blocking lipid flow. Glycolytic and oxidative metabolisms can both be altered by doxycycline, while the carbohydrate metabolism can be affected by mebendazole. Devimistat already showed antitumor activity in xenograft mouse models of human colorectal cancer, enhancing therapeutic efficacy and preventing irinotecan-triggered p53 stabilization, making it a promising candidate to support antineoplastic therapy [175]. Other drugs for non-oncological use have shown off-label efficacy in PDAC, modulating proliferative arrest [176]. When drug targets, such as *GLUT-1*, were knocked out, a strong growth-inhibiting effect on PDAC biomass was observed, resulting in a no-growth phenotype [177].

## 5. Conclusions

Here, we discuss the metabolic profile of PDAC and its implications in terms of clinical outcomes. Blocking anabolic and catabolic processes is able to reduce PDAC progression, validating the plausible hypothesis that PDAC relies on metabolic reprogramming. Although the findings presented here identify metabolic processes as a potential target for this tumor, the translation of this approach to the clinic is slow, and clinical trials investigating metabolic reprogramming in PDAC are still few and far between. Exploring the metabolic landscape could lead to the development of innovative therapeutic strategies, increasing the chances of successful treatment and survival.

## Figures and Tables

**Table 1:** List of clinical trials for PDAC’s metabolic investigation (source https://clinicaltrials.gov/ accessed on 29 June 2022; Condition or disease: PDAC Pancreatic Ductal AdenoCaricnoma and other terms: Metabolism.

Identifier ID	Study Title	Conditions	Interventions
NCT05296421	Investigating Targetable Metabolic Pathways Sustaining Pancreatic Cancer	Primary	Procedure: Biopsy, Therapeutic Conventional Surgery Other: Uniformly-labeled [13C] glucose
NCT04565327	Hyperpolarized 13C Pyruvate MRI for Treatment Response Assessment in Patients With Locally Advanced or Metastatic Pancreatic Cancer	Primary	Drug: Hyperpolarized Carbon C 13 Pyruvate, Procedure: Magnetic Resonance Imaging (MRI)
NCT04862260	Cholesterol Disruption in Combination With FOLFIRINOX in Patients With Metastatic Pancreatic Adenocarcinoma	Primary and Metastatic	Drug: Cholesterol metabolism disruption
NCT02978547	The Effects of Neoadjuvant Metformin on Tumor Cell Proliferation and Tumor Progression in Pancreatic Ductal Adenocarcinoma	Primary	Drug: Metformin Hydrochloride 500 Mg Tablet
NCT05254171	Study of Nab-Paclitaxel and Gemcitabine With or Without SBP-101 in Pancreatic Cancer	Primary	Drug: SBP-101, Nab-paclitaxel, Gemcitabine and Placebo
NCT03450018	A Study of SLC-0111 and Gemcitabine for Metastatic Pancreatic Ductal Cancer in Subjects Positive for CAIX	Metastatic	Drug: SLC-0111, Gemcitabine Injection
NCT05132244	Monitoring and Managing Glucose Levels in People With Pancreatic Cancer	Primary	Procedure: Endocrinologist-directed target blood glucose level 4–10 mmol/L using data from a continuous glucose monitor (CGM). Other: Standard Care
NCT04915417	Neoadjuvant Stereotactic Ablative Radiotherapy for Pancreatic Ductal Adenocarcinoma	Primary	Radiation: Stereotactic Ablative Body Radiotherapy (SABR)
NCT04662879	Early Detection Initiative for Pancreatic Cancer	Primary	Other: Enriching New-onset Diabetes for Pancreatic Cancer (ENDPAC) score. Other: Abdominal imaging
NCT03525392	Study to Evaluate the Safety and Activity (Including Distribution) of 177Lu-3BP-227 in Subjects With Solid Tumors Expressing Neurotensin Receptor Type 1.	Primary	Drug: 177Lu-3BP-227 (also called 177Lu-IPN01087)
NCT03410030	Trial of Ascorbic Acid (AA) + Nanoparticle Paclitaxel Protein Bound + Cisplatin + Gemcitabine (AA NABPLAGEM)	Primary	Drug: Ascorbic Acid, Paclitaxel protein-bound, Cisplatin, Gemcitabine
NCT04245644	Efficacy of Chemopreventive Agents on Disease-free and Overall Survival in Patients With Pancreatic Ductal Adenocarcinoma: The CAOS Study (CAOS)	Primary	Behavioral: use of targeted drugs such as aspirin, B-Blockers, Metformin, ACE-inhibitors, Statins
NCT03374852	CPI-613 in Combination With Modified FOLFIRINOX in Patients With Locally Advanced Pancreatic Cancer	Primary	Drug: CPI-613 Drug: mFOLFIRNOX

## Abbreviations

Alpha-ketoglutarate (αKG); cholecystokinin (Cck); extracellular matrix (ECM); FA synthase (FASN); fatty acid (FA); glutamate-ammonia ligase (GLUL); glucose transporters (GLUTs); hormone-sensitive lipase (HSL); hypoxia inducible factor 1 alpha (HIF-1α); lipid droplet (LD); Low-density lipoprotein receptor (LDL-R); Monocarboxylate transporters (MCTs); Next-generation sequencing (NGS); nicotinamide adenine dinucleotide (NADH); nicotinamide phosphoribosyltransferase (Nampt); pancreatic ductal adenocarcinoma (PDAC); pancreatic cancer stem cells (PCSCs); pancreatic intraepithelial neoplasia (PanIN); pancreatic stellate cells (PSCs); pentose phosphate pathway (PPP); ribulose-5-phosphate isomerase (RPIA); sodium-glucose transporter (SGLTs); tricarboxylic acid cycle (TCA); ubiquitin specific peptidase 25 (USP25).
